# Environmental, climatic and host population risk factors of human cystic echinococcosis in southwest of Iran

**DOI:** 10.1186/s12889-020-09638-w

**Published:** 2020-10-27

**Authors:** Mohammad Amin Ghatee, Koorosh Nikaein, Walter Robert Taylor, Mehdi Karamian, Hasan Alidadi, Zahra Kanannejad, Faezeh Sehatpour, Fateme Zarei, Gholamreza Pouladfar

**Affiliations:** 1 Cellular and Molecular Research Center, Yasuj University of Medical Sciences, Yasuj, Iran; 2 Medical Parasitology and Mycology Department, Yasuj University of Medical Sciences, Yasuj, Iran; 3 Student Research Committee, Yasuj University of Medical Sciences, Yasuj, Iran; 4 Mahidol Oxford Tropical Medicine Research Unit, Bangkok, Thailand; 5 Centre for Tropical Medicine and Global Health, Nuffield Department of Medicine, University of Oxford, Oxford, UK; 6 Department of Parasitology and Mycology, School of Medicine, Zanjan University of Medical Sciences, Zanjan, Iran; 7 Allergy Research Center, Shiraz University of Medical Sciences, Shiraz, Iran; 8 Student Research Committee, Shiraz University of Medical Sciences, Shiraz, Iran; 9 Professor Alborzi Clinical Microbiology Research Center, Department of Paediatrics, Nemazee Teaching Hospital, School of Medicine, Shiraz University of Medical Sciences, Shiraz, Iran

**Keywords:** Cystic echinococcosis, GIS, Climate, Livestock, Environment

## Abstract

**Background:**

Cystic echinococcosis (CE), a worldwide zoonotic disease, is affected by various biological and environmental factors. We investigated dog/livestock populations, climatic and environmental factors influencing the distribution of human CE cases in Fars province, southwest Iran.

**Methods:**

We mapped the addresses of 266 hospitalised CE patients (2004–2014) and studied the effects of different temperature models, mean annual rainfall and humidity, number of frosty days, slope, latitude, land covers, close proximity to nomads travel routes, livestock and dog densities on the occurrence of CE using geographical information systems approach. Data were analyzed by logistic regression.

**Results:**

In the multivariate model predicting CE, living in an urban setting and densities of cattle and dogs were the most important CE predictors, sequentially. Dry (rained) farm, density of camel and sheep, close proximity to nomads travel routes, humidity, and slope also were considered as the determinants of CE distribution, when analyzed independently. Slope had a negative correlation with CE while temperature, frost days and latitude were not associated with CE.

**Conclusions:**

In our study, an urban setting was the most important risk factor and likely due to a combination of the high density of key life cycle hosts, dogs and livestock, a large human susceptible population and the high number of abattoirs. Farmland and humidity were highly suggestive risk factors and these conditions support the increased survival of *Echinococcus granulosus* eggs in the soil. These findings support the development of strategies for control of disease. More research is needed test optimal interventions.

## Background

The larval stage of the cestode parasite *Echinococcus granulosus* sensu *lato* (*E. granulosus* s. l.) causes cystic echinococcosis (CE) also known as cystic hydatid disease, a chronic parasitic zoonosis of humans and domestic and wild mammals. CE is a disease of poverty and usually occurs in herding communities [1, 2]. Canids are the definitive hosts of *E. granulosus* and herbivores (e.g. sheep, goats, cattle) are the main intermediate hosts. Humans acquire CE by accidentally ingesting *E. granulosus* eggs in food, water, or contaminated soil [3]. After ingestion, *Echinococcus* eggs hatch and embryos are released in the small intestine. Primary larval penetration through the mucosa leads to blood borne distribution to various body organs [4] with most cysts developing in the liver (70%) and lungs (20%) due to capillary filtration [5]. Other affected organs include the spleen, heart, brain, kidney, peritoneum and bone [6]. The clinical manifestations of CE are broad, ranging from clinically silent cysts [7] to anaphylactic shock caused by the rupture of cysts and spillage of their contents [2].

CE is a worldwide public health problem, with highly endemic areas in some regions of South America, North Africa, China, and the Middle East, including Iran. Conventional livestock husbandry and widespread close contact between people and animals are key factors in the hyper endemicity of CE in Iran, where 1% of all surgeries are linked to CE [8–10].

Within the *E. granulosus s. L. complex*, there are 10 genotypes (G1–G10) and four species. The identification of species and strains is important for CE control programs, for disease prevention and epidemiological tracking of cases [11]. In Iran, G1, G2, G3 and G6 genotypes have been reported from humans and livestock including sheep, goats, camels and cattle, while the G7 genotype is isolated only from sheep and goats [12–15]; G1 is the most frequent followed by G6 and in Fars province, southwestern Iran, only G1 and G6 genotypes have been found in the hosts [12, 16].

Risk factors for CE in endemic regions include the presence of free roaming dogs, being a dog owner, slaughtering livestock at home or in inadequately supervised slaughterhouses [17]. Moreover, the CE cycle is extensively dependent to environmental and climatic factors due to their effects on the survival of eggs and on the living conditions of humans and livestock. *E. granulosus* eggs passed in dog faeces may survive 3 weeks at 30 °C, 4.5 weeks at 10–21 °C, and 32 weeks at 6 °C in water and damp sand and for several months in green pastures and gardens [18]. Also, livestock and stray dog’s life as the main intermediate and final hosts are extensively affected by environmental and climatic conditions.

Geographical information systems (GIS) are computer-based approaches for integrating and analyzing geo-spatial data. They are valuable tools for studying the effects of environmental and climatic factors on disease occurrence [19], predicting disease trends and distribution, and modelling the control of diseases over time [20, 21]. GIS have been used recently to study different parasitic diseases such as leishmaniasis and malaria in Iran [22, 23].

GIS-based studies on human CE are few in different countries where some geo-climatic factors have not been even approached so far. Fars is the largest province in southwest Iran and one of the highest endemic areas of CE with human infection rate up to 13.7% [24], but GIS analyses have not been used to study CE distribution and role of the main geo-climatic factors on CE in this region. Herein, we comprehensively report on the host, environmental and climatic factors affecting human CE in Fars.

## Methods

### Study area

**Fig. 1:**
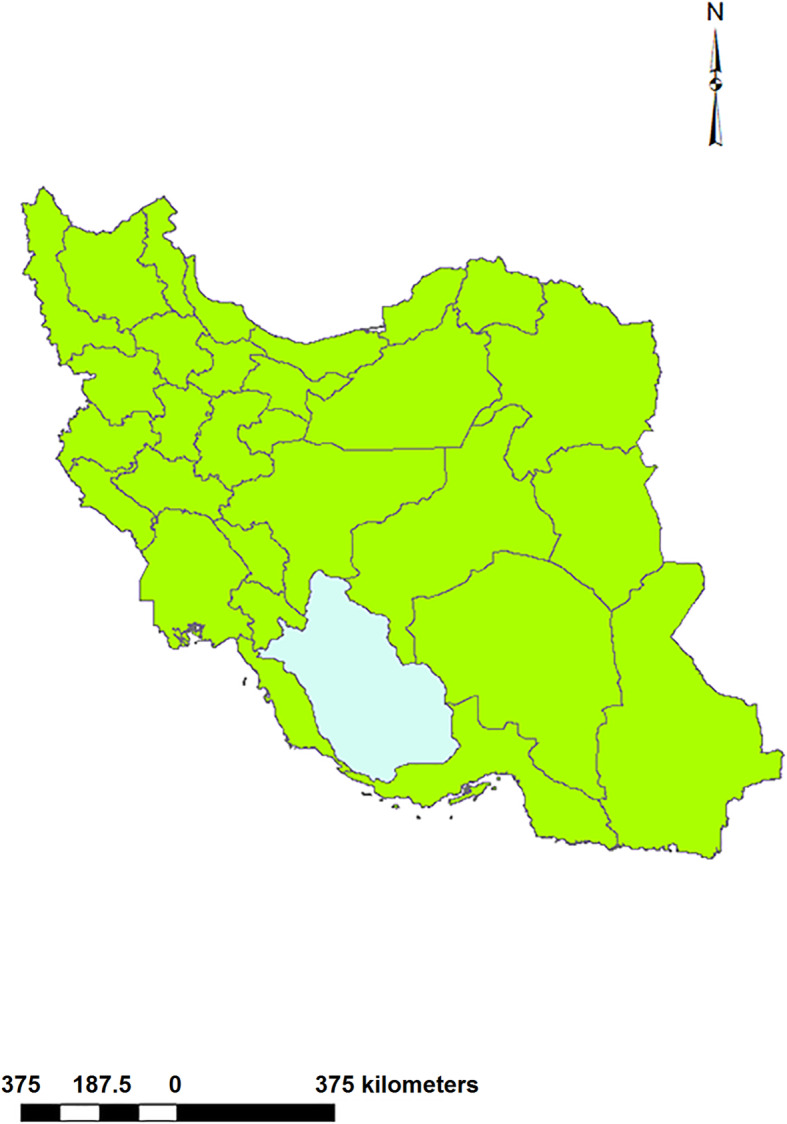
Map of Iran showing Fars province in the southwest. Note: Map was created with ArcMap from ArcGIS 10.1

Fars Province lies in southwest Iran, between longitude 27° 31′N to 31° 42′N and latitude 50° 37′E to 55° 38′E, and covers an area of about 122,608 km^2^ (Fig. 1). The total population of Fars is about 4.6 million with a male to female ratio of 1: 1.03. Administratively, it is divided into 24 counties and Shiraz is the provincial capital. The region encompasses a variety of landscapes from dense forests to bare plains and elevations of 115 to 3115 m above sea level; the Zagros mountain range stretches from the northwest to the southeast and covers 70% of Fars. The population consists of urban, suburban and rural dwellers and there is a substantial nomadic population; the latter come from different clans who spend much of their lives herding their livestock and dogs between their winter (Qishlag) and summer quarters (Yailaq) along well established routes.

### Data collection

From 2004 to 2014, we obtained the addresses of 266 patients who were treated for confirmed CE at hospitals of Fars province. All data were retrieved from patient’s records and gathered in Professor Alborzi Clinical Microbiology Research Center in Namazi hospital in Shiraz.

### Geospatial data

The CE patients’ residences were recorded on the point shape file map of Fars province, according to the latitudes and longitudes of the villages and cities from which the patients were drawn. The digital elevation model (DEM) raster layer and the province and land cover vector layers were acquired from the Department of Natural Resources in Fars province. The slope raster map was drawn through spatial analyst tool, based on DEM, by computing the maximal rate of variation in value between each cell and its adjacent cells and the land cover layer shows spatial data on the different physical characteristics of the surface of the province.

For the period 2004 to 2014, temperatures, humidity and evaporation data and the frequencies of rainy and frosty days, from 18 synoptic meteorological stations, and rainfall data from 86 rain-gauge stations were acquired from the Fars Province Weather Bureau. From these data, the mean annual temperature (MAT), maximum mean annual temperature (maximum MAT), minimum mean annual temperature (minimum MAT), mean annual humidity (MAH), mean annual rainfall (MAR), mean annual evaporation (MAE) and mean rainy and frosty days were calculated. After testing various interpolation methods, the annual iso-hydral, iso-humid and frost days raster layers were generated using the Kriging interpolation method and iso-thermal, iso-evaporation and rainy days raster layers using the tension based Spline interpolation model with a resolution grid of 2 × 2 km.

### Nomad travel routes risk maps

The vector layer of nomads’ travel routes (NTR) was obtained from the Nomads Affairs Administration of Fars province. Two risk maps were developed by unifying the buffers (2 and 5 km) that made around the routes along which the nomads migrate between Yailaq and Qishlag to investigate the association between nomad population and CE occurrence.

### Livestock and dog densities

The livestock and dog data, including geographical coordinates of livestock sheds and the number of sheep, goats, cows, camels and dogs in each shed, were obtained from the Fars province Veterinary Bureau to generate a livestock shed point shapefile layer and animal density raster layers using the Kernel (method) density method.

### Geospatial analysis

ArcGIS version 10.1 (http://www.esri.com/arcgis) was used to analyze geospatial and climatic data. The provincial villages and cities point shape file layer was extracted with the raster layers. The identity tool was used to compute the geometric intersection of the layer obtained from the extraction of all raster layers with NTR hazard (polyline) and land cover (polygonal) vector layers to develop the final layer in which each point represented properties of all the overlapped identity features from the above-mentioned raster and vector layers. The attribute of this layer was converted to an excel format for statistical analysis. All maps were provided by Ghatee et al. by using ArcMap from ArcGIS 10.1.

### Statistical analysis

After the spatial description of CE patients in Fars province, the association between disease and possible the risk factors (i.e. human and animal populations, climate, and environment) was investigated. Accordingly, residential points data including CE reported and non-reported villages and cities were extracted from final province villages/cities point layer and analyzed using univariate and multivariate Enter logistic regression models. The analyses were performed using SPSS version 21.

## Results

### Geo-climatic distribution of infected points

A total of 266 CE patients were identified from hospital records who lived in 134 points in the province, representing 1.6% of the 8182 villages and cities in Fars. Villages/cities with report of cases of CE were distributed in different parts of province but three clusters were evident in central, western and southeastern areas of Fars with most cases coming from the central region. One hundred thirty nine and 127 of patients were from cities and rural points, respectively.

**Fig. 2:**
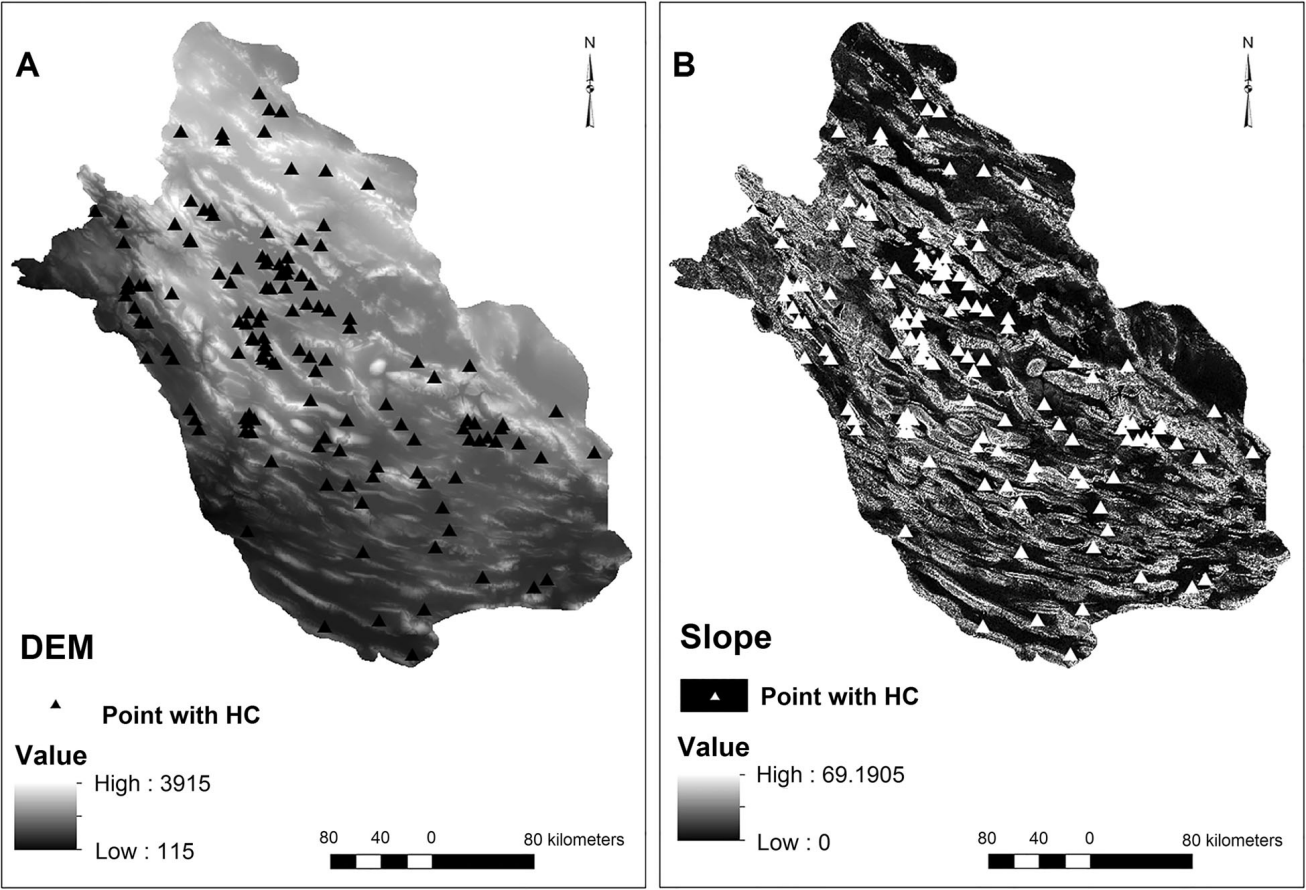
Digital elevation model (**a**) and Slope (**b**) maps. Infected points were shown by triangle symbol. No cases of hydatid cyst were reported on the slopes higher than 43 degrees. The digital elevation model (DEM) raster layer was acquired from the Department of Natural Resources in Fars province, Iran. Slope raster layer was generated based on the DEM layer. Note: Maps were created with ArcMap from ArcGIS 10.1 by our team

**Fig. 3:**
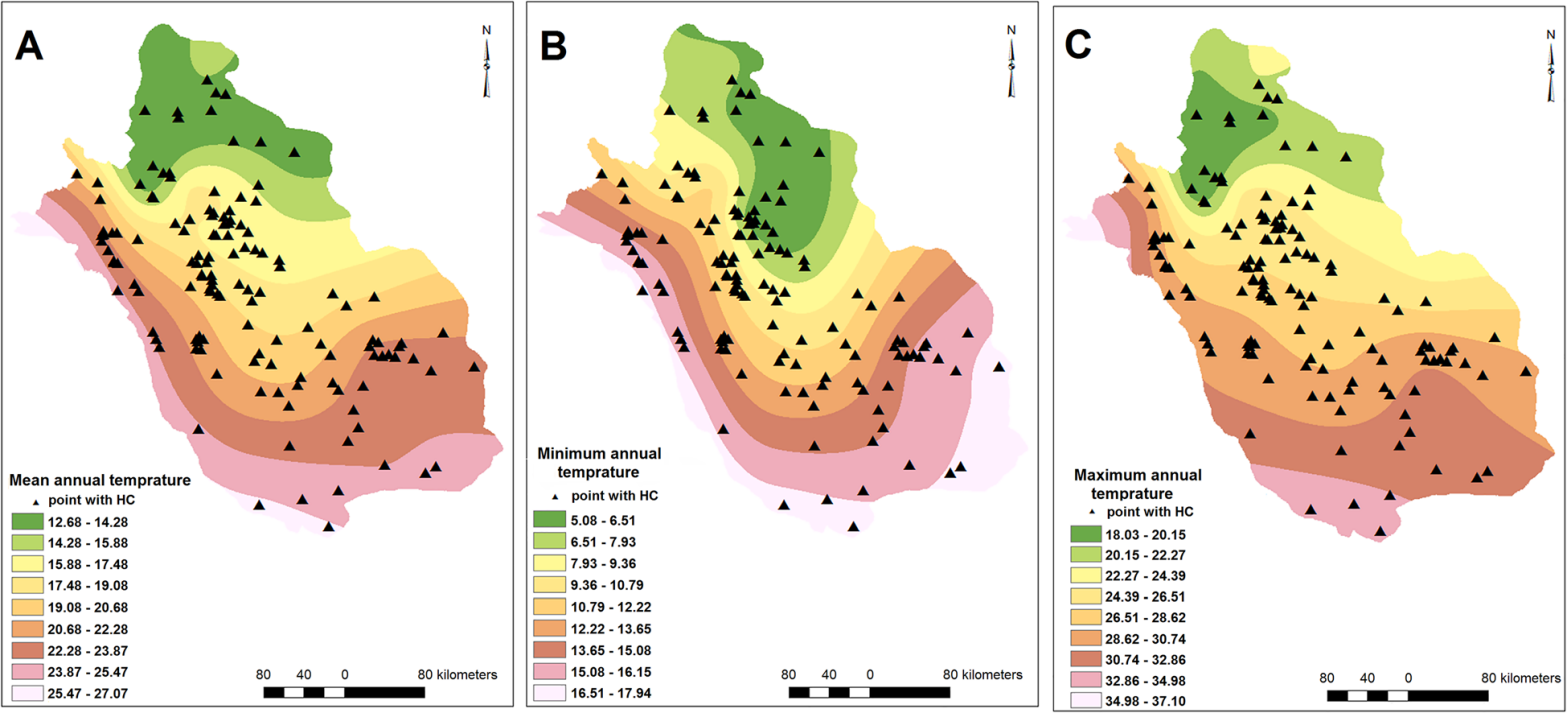
Isothermal models rasters; Mean annual temperature (**a**), Minimum mean annual temperature (**b**), and Maximum mean annual temperature (**c**). Infection points were distributed in different ranges of temperature and no association was detected between different models of temperature and hydatid cyst distribution in Fars province. Note: Maps were created with ArcMap from ArcGIS 10.1. The mentioned layers and maps were generated by our team based on the meteorological data

**Fig. 4:**
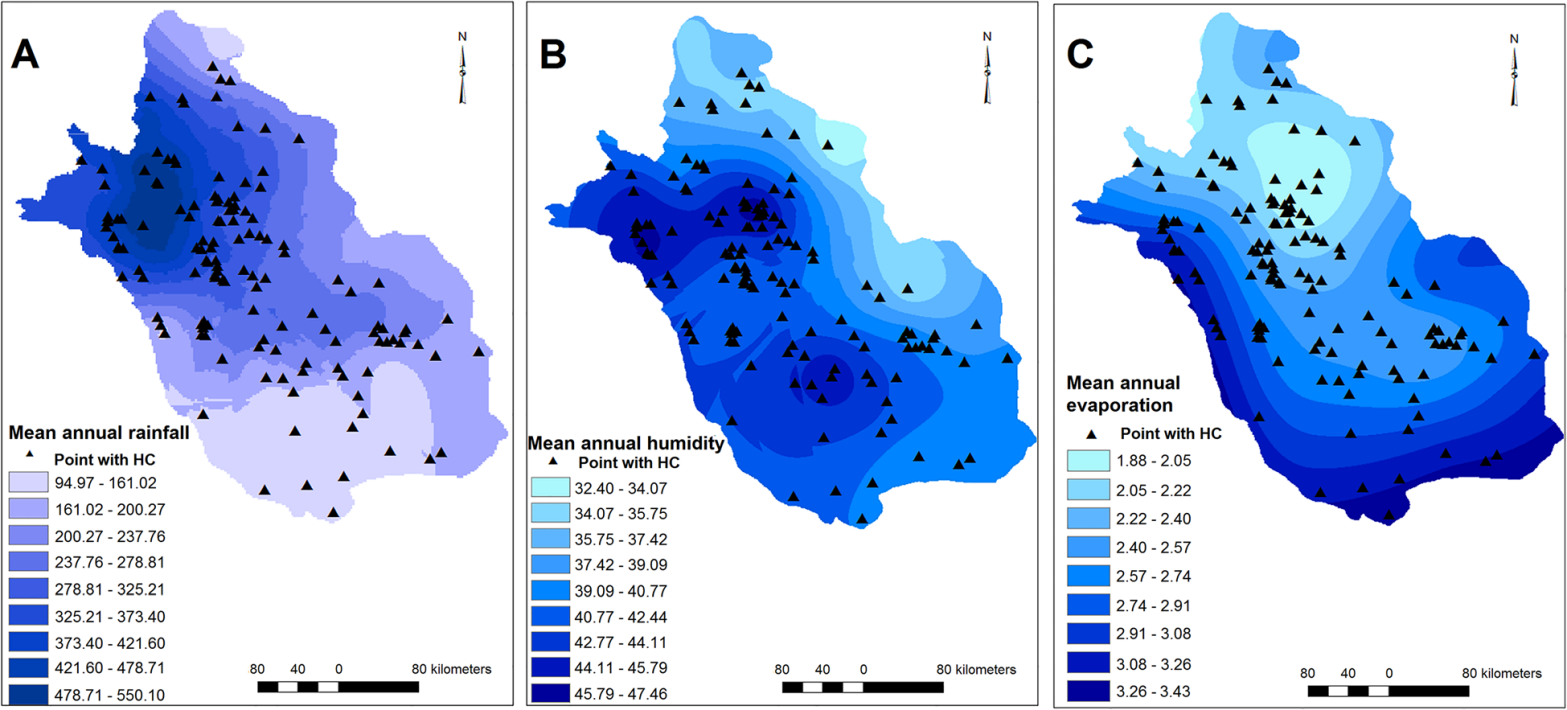
Rainfall (MAR) (**a**), Humidity (MAH) (**b**) and evaporation (MAE) (**c**) ratser models. Note: Maps were created with ArcMap from ArcGIS 10.1 and by our team based on the meteorological data

**Fig. 5:**
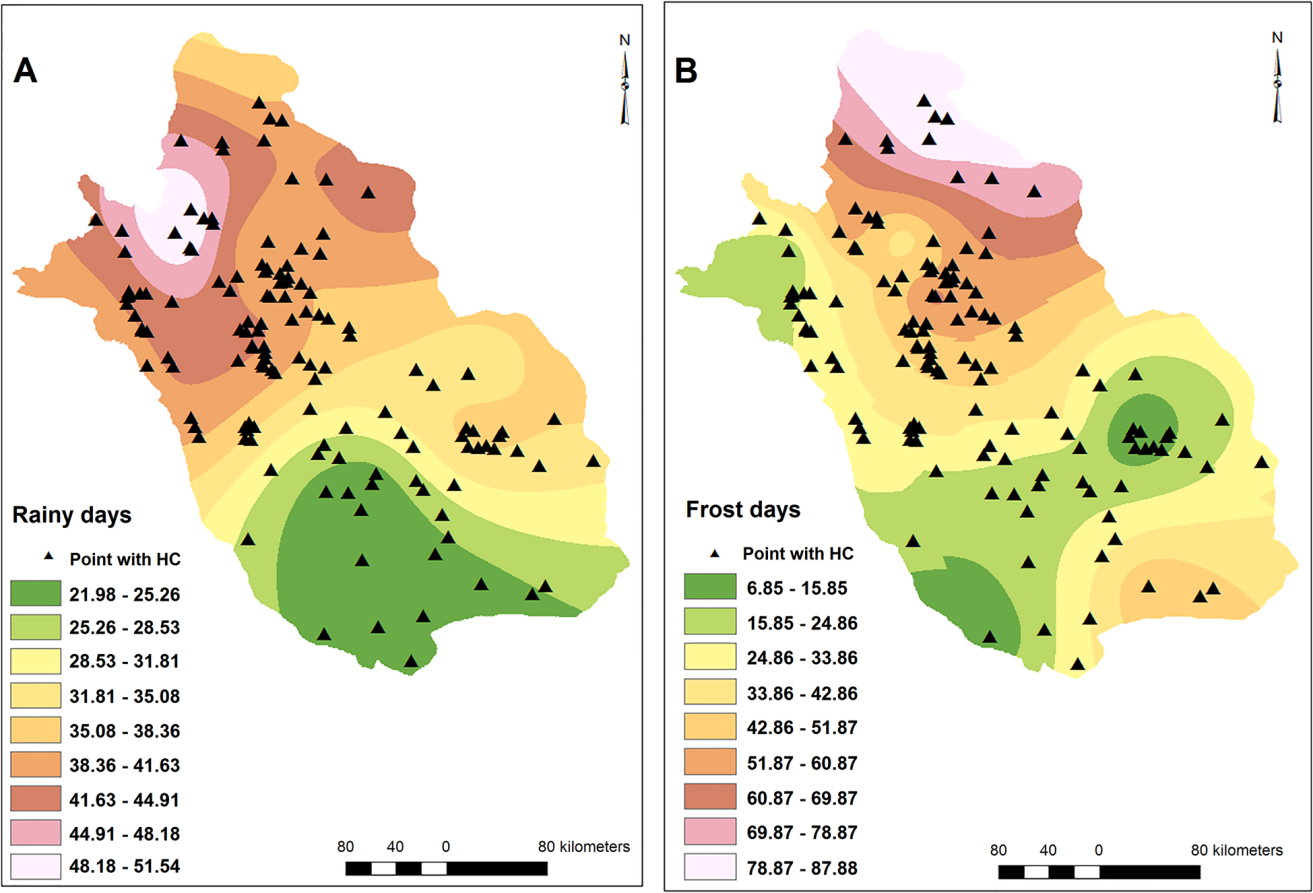
The raster maps of the number of rainy days (**a**) and frosty days (**b**) and the distribution of infection points. Hydatid disease was not associated with these factors in Fars province. Note: Maps were created with ArcMap from ArcGIS 10.1 and by our team based on the meteorological data

**Fig. 6:**
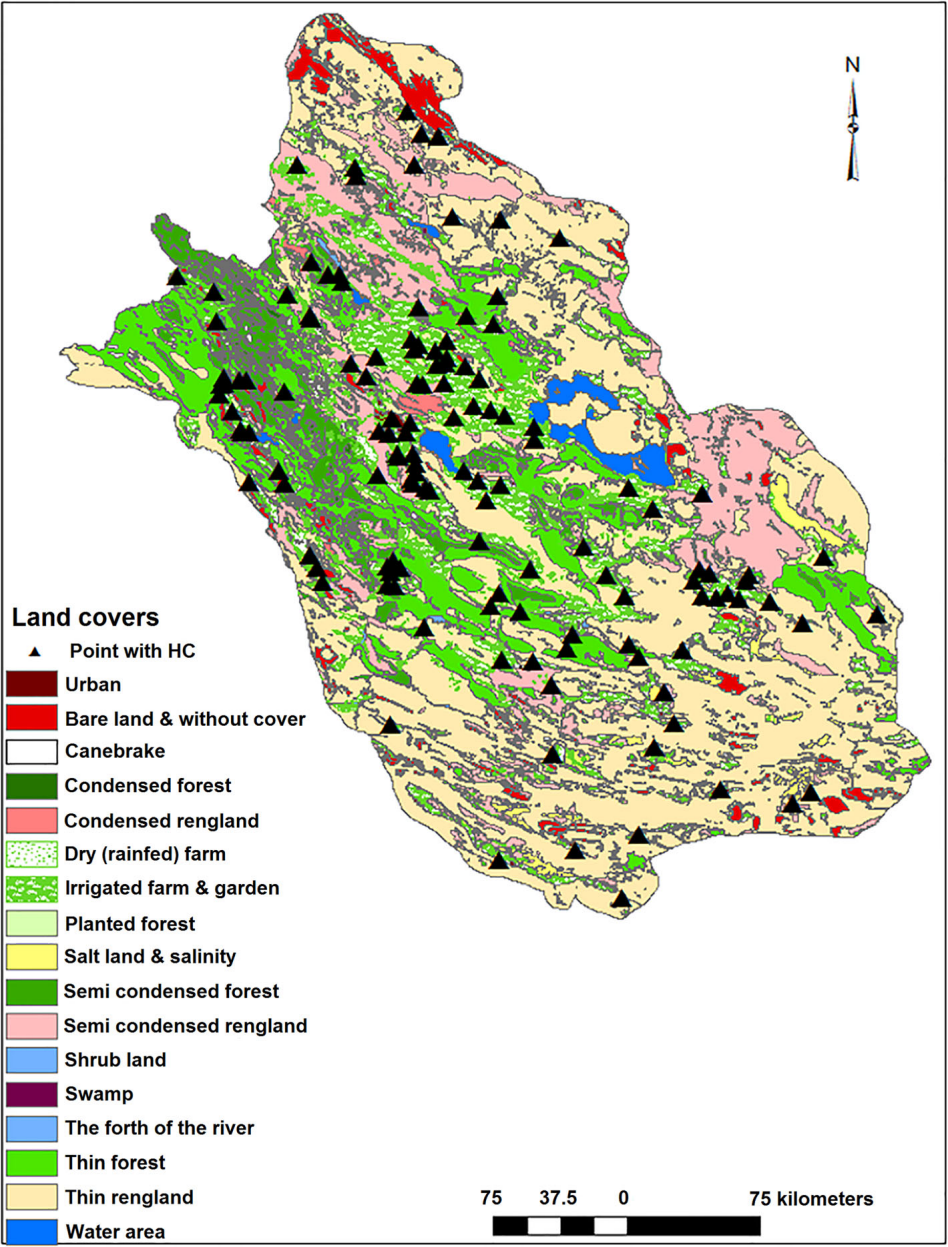
Land cover vector map of Fars province. A notable number of infection points were distributed in farmlands and urban areas. Urban areas are marked by triangles. Note: Map was created with ArcMap from ArcGIS 10.1. The land cover vector layers were acquired from the Department of Natural Resources in Fars province, Iran

Similarly, villages/cities with CE cases reported were distributed in regions with different climatic and environmental conditions. The elevation and slope varied from 279 to 2365 m and 0–43 degrees, respectively (Fig. 2). MAT, minimum and maximum MAT ranged 12.8–25.6 °C, 5.1–17.1 °C and 18.1–34.1 °C, respectively (Fig. 3). MAR ranged from 115 to 540 mm, minimum and maximum air humidity were 33 and 67%, respectively, and evaporation levels were between 1889 and 3385 mm among villages/cities with CE cases reported (Fig. 4). The number of rainy days and frosty days ranged 22–51 and 7–86 days, respectively for these points (Fig. 5). The majority of infected points were found different types of terrain and the highest ratio of infected point frequency was found in urban areas followed by dry and irrigated farmlands (Table 1, Fig. 6).

**Table 1:** The distribution of CE infection points and all points and their ratios in various land covers of Fars province

Land cover	Infected points per feature (%)	All points per feature (%)	Ratio
Urban	14.9	1.3	11.46
Condensed and Semi-condensed forest	0.7	5.2	0.13
Sparse forest	2.2	10.2	0.22
Condensed and Semi-condensed rangeland	4.5	9.3	0.48
Thin rangeland	12.7	27	0.47
Dry (rainfed) farm	3	2.1	1.43
Irrigated farm	59	41.6	1.42
Salt land, salinity and water area	3	3.4	0.88
Total	100	100

### Univariate logistic regression

#### Environmental and climatic factors

The logistic regression for single variable model showed irrigated, dry farms and urban land covers, slope and MAH effect on CE occurrence in southwest Iran. Among them, land covers showed the highest effect on the occurrence of CE; Urban (*p* value < 0.001, OR = 97.01), dry farm (p value = 0.038, OR = 10.23) and irrigated farm (*p* value = 0.022, OR = 10.03). Ratio of villages/cities with CE cases reported to all points was higher than 1 in the above-mentioned land covers (Table 2). Increasing slope protected against CE (p value = 0.002, OR = 0.935) while humidity showed positive correlations with CE (p value< 0.001, OR = 1.13). Increase of one unit of each aforesaid determinant increased and decreased the possibility of CE occurrence by 13 and 6.5%, respectively (Fig. 2, Fig. 4). Other geo-climatic factors including MAT, maximum MAT, minimum MAT, MAR, elevation, and the number of frost and rainy days had no significant effect on the occurrence of CE in our study (Table 2).

**Table 2:** Results of Univariate logistic regression model for evaluation of effect of geo-climatic factors, close proximity to NTR and animal densities on CE in Fars province, southwest Iran

Variable	***P***-value	OR	CI
Land cover			
Condensed and semi condensed forest	< 0.001^a^		
Sparse forest	0.717	1.520	0.158–14.655
Condensed and semi-condensed rangeland	0.263	3.358	0.403–27.987
Thin rangeland	0.250	3.270	0.434–24.636
**Dry (rainfed) farm**	**0.038**	**10.230**	**1.135–92.207**
**Irrigated farm**	**0.022**	**10.039**	**1.393–72.339**
**urban**	**< 0.001**	**97.011**	**12.849–732.461**
Salt land, salinity and water area	0.103	6.206	0.690–55.818
MAT	0. 8	1.006	0.959–1.056
MinMAT	0.398	0.980	0.934–1.027
MaxMAT	0.695	1.009	0.966–1.054
MAR	0.157	1.001	1.000–1.003
**MAH**	**< 0.001**	**1.136**	**1.068–1.209**
MAE	0.283	1.000	0.999–1.000
Mean rainy day	0.512	1.008	0.984–1.032
Elevation	0.058	1.000	0.999–1.000
**slope**	**0.002**	**0.935**	**0.897–0.975**
Frost day	0.645	1.002	0.993–1.011
**2 km buffer around NTR**	**< 0.001**	**2.087**	**1.482–2.937**
**5 km buffer around NTR**	**< 0.001**	**2.191**	**1.501–3.198**
**Sheep density**	**< 0.001**	**1.006**	**1.003–1.009**
**Cattle density**	**< 0.001**	**1.038**	**1.029–1.047**
**Camel density**	**0.029**	**4.046**	**1.150–14.241**
**Dog density**	**< 0.001**	**3.121**	**2.104–4.630**

^a^Condensed and semi-condensed forest was fitted as reference group for landcovers. *CI* Confidence interval, *OR* Odd’s ratio, *MAT* Mean annual temperature, *MinMAT* Minimum mean annual temperature, *MaxMAT* Maximum mean annual temperature, *MAR* Mean annual rainfall, *MAH* Mean annual humidity, *MAE* Mean annual evaporation, *NTR* Nomad travel route

### Effect of close proximity to NTR

**Fig. 7:**
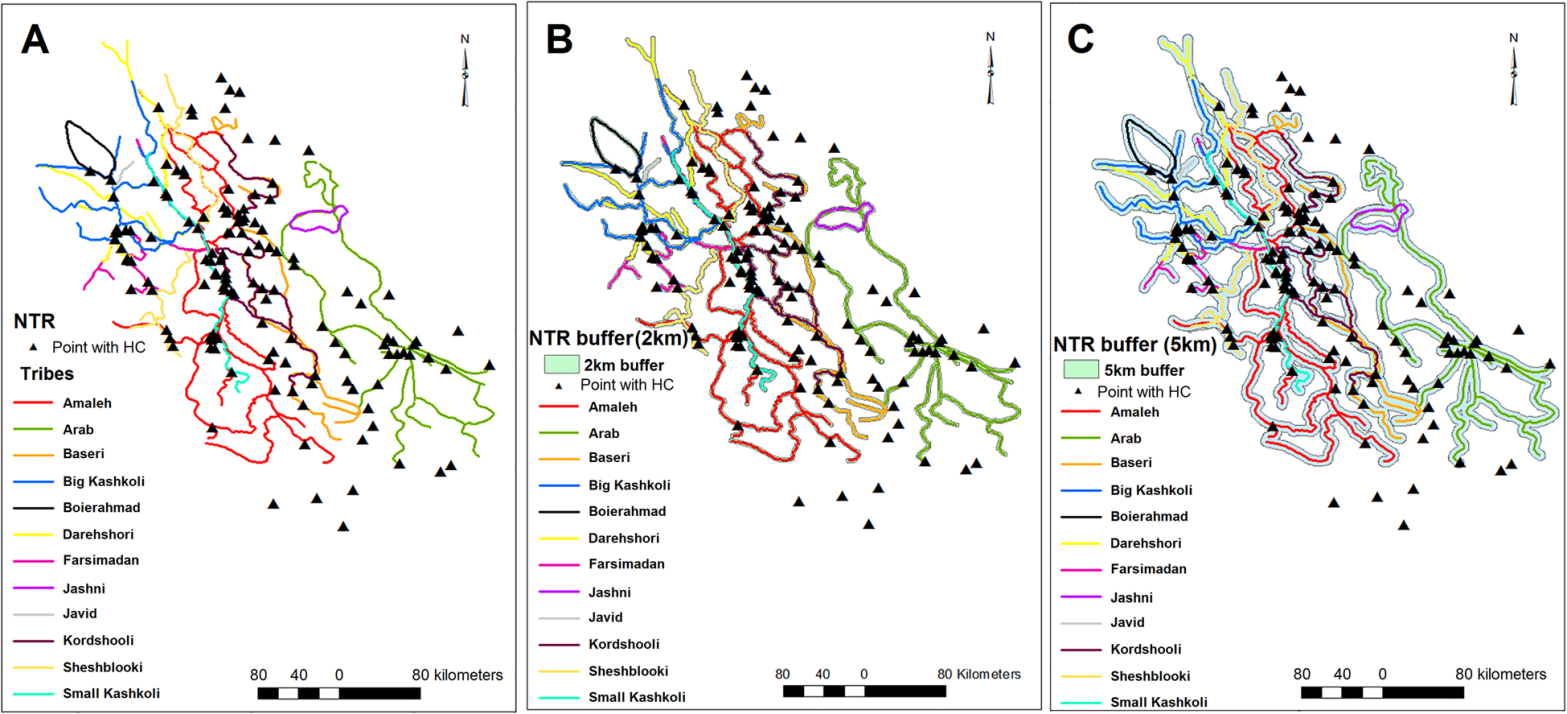
Maps of nomad travel routes (**a**) and 2 km (**b**) and 5 km (**c**) buffers around nomad travel routes. Most of the infection points are located close to these routes. Note: Maps were created with ArcMap from ArcGIS 10.1. The vector layer of nomads’ travel routes (NTR) was obtained from the Nomads Affairs Administration of Fars province, Iran

Close proximity to NTR also affects on the occurrence of CE in the Fars province (Table 2). Presence of village/city points in 2 km and 5 km buffers increased the possibility of CE occurrence by 208% (*p* value< 0.001, OR = 2.087) and 219% (*p* value< 0.001, OR = 2.191), respectively. From 134 points with report of CE, 66 and 96 points were distributed in 2 km and 5 km buffers around NTR, respectively (Table 2, Fig. 7).

### Livestock and dog densities

**Fig. 8:**
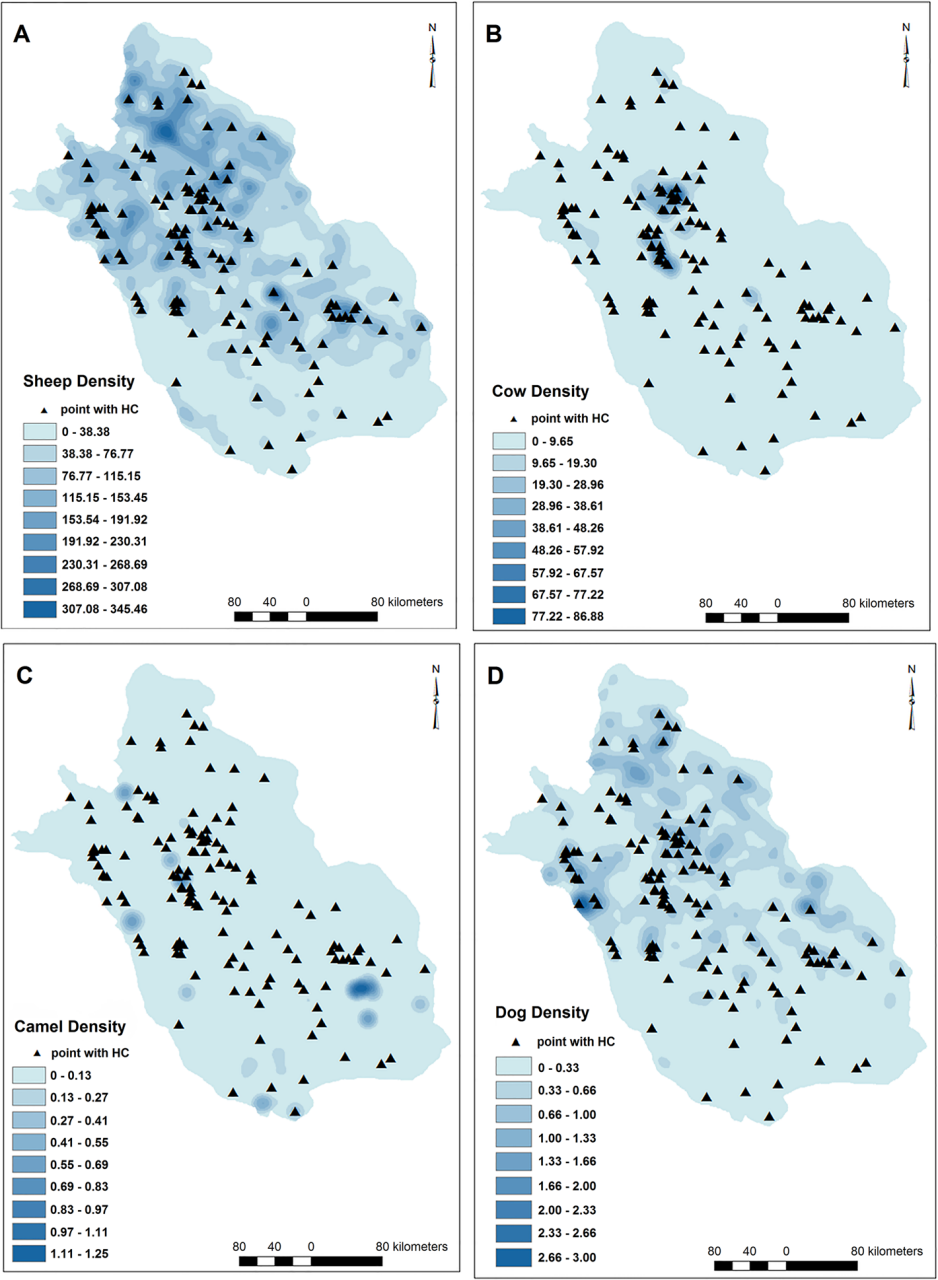
The raster maps of the densities of sheep or goats (**a**), cattle (**b**), camels (**c**) and dogs (**d**). Note: Maps were created with ArcMap from ArcGIS 10.1 and by our team based on the livestock data

The density of sheep/goat (*p* value< 0.001, OR = 1.006), cattle (*p* value< 0.001, OR = 1.038), dog (*p* value< 0.001, OR = 3.121) and camel (*p* value = 0.029, OR = 4.046) in km^2^ significantly influenced CE distribution and increased the possibility of disease occurrence (Table 2). Density of camel was the most effective determinant among the animals on the CE occurrence (Table 2, Fig. 8).

### Multivariate logistic regression model

Of the 16 tested variables (Table 3), only an urban setting (*p* value < 0.001, OR = 67.646) and density of dogs (*p* value = 0.037, OR = 1.944) and cattle (*p* value = 0.007, OR = 1.203) were significant explanatory variables whilst irrigated farms were borderline significant and there were trends for dry farmlands and humidity. Urban land cover was the most important predictive determinant in this model.

**Table 3:** Results of enter multivariate logistic regression model analysis

Variable	***P***-value	OR	CI
Land cover			
Condensed and Semi condensed forest	< 0.001		
Mean humidity	0.072	1.073	0.994–1.158
Slope	0.881	1.003	0.964–1.043
Sparse forest	0.789	1.363	0.140–13.264
Condensed and Semi-condensed rangeland	0.276	3.298	0.385–28.216
Thin rangeland	0.213	3.368	0.474–28.631
Dry (rainfed) farm	0.081	7.468	0.782–71.332
Irrigated farm	0.050	7.460	0.969–57.431
**Urban**	**< 0.001**	**67.646**	**8.371–546.632**
Salt land, salinity and water area	0.144	5.205	0.570–47.571
2 km buffer around NTR	0.156	1.384	0.883–2.168
5 km buffer around NTR	0.981	1.006	0.595–1.701
Livestock density	0.558	0.440	0.028–6.856
Sheep density	0.906	1.000	0.996–1.004
**Cattle density**	**0.007**	**1.203**	**1.006–1.041**
Camel density	0.801	1.251	0.220–7.103
**Dog density**	**0.037**	**1.944**	**1.040–3.636**

## Discussion

We have shown that the occurrence of CE in Fars province, southwestern Iran, was affected mainly by an urban setting and the densities of cattle and dogs. Our results also suggest that different types of farmland, humidity, density of camel and sheep, proximity to nomadic travel routes, and slope also affected the distribution of CE when evaluated independently from other factors.

An urban setting was the most important factor in the distribution of CE and this is related to the size of urban populations, the industrial herding of large flocks of sheep and goat and, the presence of a large number of stray dogs in the suburb of urban areas. Moreover, populations living in suburbs of cities and metropolitans mostly have lower socioeconomic conditions, and usually breed livestock and shepherd dogs in the suburbs and periurban areas [25], where livestock is slaughtered in abattoirs, butchers’ shops and peoples’ homes. It is well established that dogs may become infected by eating the offal of slaughtered livestock in nearby local butcher shops and abattoirs or from houses in which sheep are killed for household consumption [26, 27]. Torgerson et al. (2003) showed that a higher prevalence of infection was found in dogs which are closely associated with livestock in comparison to dogs kept as house pets [28]. This may have been important in our study but we did not examine this. Consistent with the distribution of zoonotic related leishmaniasis in southwest Iran, where dogs are the most important reservoirs, urban areas are also an important determinant of this disease [29].

Densities of cattle and dogs were the next factors explaining the distribution of human CE. Canids especially dogs, as the definitive hosts of *E. granulosus*, are necessary for the continued transmission of CE. Dogs become infected principally by consuming infected offal of slaughtered ruminants left lying around in insanitary and substandard abattoirs. In some provinces of Iran, it is stray dogs that have the highest rate of *Echinococcus* infection (19.1%) among the canids [30] and studies also reported high infection rates in shepherd dogs, up to 63.3% [27].

In our study, cattle were the most important livestock affected the occurrence of human CE in Fars province. Although the number of sheep and goats is higher than cattle in the studied areas, the CE prevalence rates in Fars are highest in cattle (11%), sheep (just under 5%) and goats (4.5%) [31]. Also, another study in northern Iran (Mazandaran province), showed that 40.1% of cattle and 37.8% of goats were infected by CE [32].

The importance of cattle may be related to the timing of their slaughter. They are slaughtered in older age than sheep and goat; therefore, potential *E. granulosus* egg exposure is longer in cattle [3]. The higher prevalence of CE in older cattle was reported in some studies which is also due to more exposure time with pastures contaminated with egg [33–35].

Farming was the other important factor that we identified due to the herding of livestock, with sheepdogs, on farmland. High soil moisture, especially in irrigated farms, supports the survival of *E. granulosus* eggs [36], and increases the probability of disease transmission to intermediate hosts. In China, Yang et al. showed that an increase in farming following deforestation was associated with an increased risk of CE in humans and intermediate herbivores [37]. In Italy, the green fodder around cattle farms was assumed as the main cause of infection of cattle with *Echinococcus* eggs [7]. Limited knowledge of hydatid disease among farmers working in these areas also plays a considerable role in the occurrence of CE in farm regions [38, 39].

In our univariate analysis, camel density was an important risk factor that was not significant in the multivariate model. However, other data from Iran support the importance of camels as intermediate hosts. Some 35% of slaughtered camels from different regions of Iran were infected with hydatid cysts [40], in southwest region including Fars province, CE prevalence rates was (70%) higher than central (64%), South (42%) and northeast (11%) regions, [41–43] indicating the high potential of camel in the transmission of *E. granulosus* in Fars province.

Close proximity to NTR was also an effective factor in the univariate model but not in the multivariate model. Nevertheless, the herding life style of nomadic populations results in close contact with sheep, goats, and dogs and ample opportunity to be infected by CE. Furthermore, on their twice annual long journeys of hundreds of kilometers, nomads pass through known areas endemic for CE and have contact with stray dogs and wild canids, exposing them and their dogs to different CE strains; moreover, serological studies have shown high seropositivity for CE in nomadic populations. In recent times, many nomadic families have changed their life-styles and have opted to settle in villages and cities near their NTRs where their presence may enhance CE transmission [29]. In Iran, Rafiei et al. reported a 14% seropositivity rate in nomads compared to 4.3% in rural inhabitants [44, 45].

Humidity was a significant factor in the univariate model and showed a strong trend for CE occurrence in the multivariate model and results in increased soil moisture, especially in winter, and this greatly increases the chances of egg survival and therefore, transmission of infection to intermediate hosts and human [46]. Wang et al. showed that the concentration of *E. granulosus* eggs was higher in the seasons with more moisture like winter [47], and Laws et al. have confirmed the greater longevity of *E. granulosus* eggs in humid conditions with survival rates of 50, 20 and 5% in 80, 60 and 25% relative humidity, respectively [48].

Slope was revealed as the factor with limiting effect with the univariate model on CE occurrence in southwest Iran, where more CE reported villages/cities were found in lower slope. Generally, rainwater carries eggs from high lands to low altitudes and lower slope regions. Also, Guislain et al. revealed eggs which were washed by rain can survive more than dry eggs [49, 50]. On the other hand, historically humans have been forming villages near the rivers, in low altitude regions which are often in low slope. The restricting effect of increase of slope on the occurrence of other parasitic diseases was reported in southwest Iran [22, 29].

Although temperature was shown as an important factor affecting the survival of *Echinococus* eggs [51], no relation between temperature and the occurrence of EC was found in current study. It could be explained by the higher effect of other mentioned factors and more important role of humidity in the survival of eggs in this mostly semi-arid region of Iran.

## Conclusions

This reasonably sized retrospective study from Fars province has shown that the density of cattle and dogs and the urban setting were strongly implicated in CE transmission whilst humidity and farmlands were highly suggestive. Univariate models showed several important factors which were not significant in the multivariate analysis like proximity to NTRs, dog, camel and sheep density and slope but these factors have been reported as important by others.

A greater understanding of the human, dog, and intermediate host and climatic factors will help CE control programmes to better devise mitigations strategies. In particular, more research is needed on the relation between abattoir location and CE and the effect of the changing lifestyle of the nomadic populations. Researcph that integrates GIS, molecular based studies and human behavior would also inform policy makers.

## Data Availability

All data were retrieved from hospital records almost all from Namazi hospital, Shiraz the main referral hospital in southwest Iran. Retrieved data were and gathered in Professor Alborzi Clinical Microbiology Research Center in Namazi hospital in Shiraz. The datasets generated and/or analysed during the current study are not publicly available due to some geographical details of cities/villages that may be used by unauthorized persons but are available from the corresponding author on reasonable request.

## Abbreviations

CE: Cystic Echinococcosis
GIS: Geographical Information Systems
DEM: Digital Elevation Model
MAT: Mean Annual Temperature
maximum MAT: Maximum Mean Annual Temperature
minimum MAT: Minimum Mean Annual Temperature
MAH: Mean Annual Humidity
MAR: Mean Annual Rainfall
MAE: Mean Annual Evaporation
TR: Nomads’ Travel Routes
*E. granulosus* s. l: Echinococcus granulosus sensu lato
